# Microbiological contamination in counterfeit and unapproved drugs

**DOI:** 10.1186/2050-6511-15-34

**Published:** 2014-06-26

**Authors:** Dieter Pullirsch, Julie Bellemare, Andreas Hackl, Yvon-Louis Trottier, Andreas Mayrhofer, Heidemarie Schindl, Christine Taillon, Christian Gartner, Brigitte Hottowy, Gerhard Beck, Jacques Gagnon

**Affiliations:** 1 AGES - Austrian Agency for Health & Food Safety, Austrian Medicines and Medical Devices Agency, Traisengasse 5, Vienna AT-1200, Austria; 2 Health Canada, 1001 St-Laurent West, Longueuil, Qc J4K 1C7, Canada

## Abstract

**Background:**

Counterfeit and unapproved medicines are inherently dangerous and can cause patient injury due to ineffectiveness, chemical or biological contamination, or wrong dosage. Growth of the counterfeit medical market in developed countries is mainly attributable to life-style drugs, which are used in the treatment of non-life-threatening and non-painful conditions, such as slimming pills, cosmetic-related pharmaceuticals, and drugs for sexual enhancement. One of the main tasks of health authorities is to identify the exact active pharmaceutical ingredients (APIs) in confiscated drugs, because wrong API compounds, wrong concentrations, and/or the presence of chemical contaminants are the main risks associated with counterfeit medicines. Serious danger may also arise from microbiological contamination. We therefore performed a market surveillance study focused on the microbial burden in counterfeit and unapproved medicines.

**Methods:**

Counterfeit and unapproved medicines confiscated in Canada and Austria and controls from the legal market were examined for microbial contaminations according to the US and European pharmacopoeia guidelines. The microbiological load of illegal and legitimate samples was statistically compared with the Wilcoxon rank-sum test.

**Results:**

Microbial cultivable contaminations in counterfeit and unapproved phosphodiesterase type 5 inhibitors were significantly higher than in products from the legal medicines market (p < 0.0001). Contamination levels exceeding the USP and EP limits were seen in 23% of the tested illegal samples in Canada. Additionally, microbiological contaminations above the pharmacopoeial limits were detected in an anabolic steroid and an herbal medicinal product in Austria (6% of illegal products tested).

**Conclusions:**

Our results show that counterfeit and unapproved pharmaceuticals are not manufactured under the same hygienic conditions as legitimate products. The microbiological contamination of illegal medicinal products often exceeds USP and EP limits, representing a potential threat to consumer health.

## Background

The counterfeiting of pharmaceuticals has been a known problem for decades. In recent years, the challenge has escalated and the numbers of counterfeit drugs have increased continuously, not only in developing but also in developed countries [1]. Available estimates on the value of the global market for counterfeit drugs are in the range of US$ 75 to US$ 200 billion, indicating the significance of the problem [1,2]. In West Africa alone, the illegal antimalarial drug market may exceed US$ 400 million [3,4]. In developed countries, life-style drugs, such as phosphodiesterase type 5 (PDE5) inhibitors used for the treatment of erectile dysfunction, seem to be the main targets for counterfeiting [5]. In the face of rising drug costs, counterfeit versions of cancer drugs and other life-saving medicines are also on the rise worldwide [6]. Overall, any medication that is in high demand is an attractive target for counterfeiters [5].

According to the definition by the World Health Organization (WHO), a counterfeit medicine is “one which is deliberately and fraudulently mislabelled with respect to identity and/or source. Counterfeiting can apply to both branded and generic products and counterfeit products may include products with the correct ingredients or with the wrong ingredients, without active ingredients, with insufficient active ingredients or with fake packaging” [7]. However, “a counterfeit drug is defined differently in different countries” [7].

Unapproved medicines are drugs sold or imported without having been granted a marketing authorisation by health authorities [8]. Unapproved drugs are often marketed as being similar to, or a foreign version of, an approved drug. Such medicines may indeed comply with the quality standards in their country of origin, but because they are not imported or sold through the legal supply chain, their origin often remains unclear and their compliance with the quality standards of the target country cannot be verified [9,10].

Consumers are generally unaware of the dangers associated with the use of counterfeit and unapproved life-style drugs. Next to treatments for erectile dysfunction, appearance-enhancing medications such as slimming pills or anabolic steroids are in high demand. While non-treatment with these drugs does not lead to detrimental health effects, their use can result in dangerous adverse effects caused by overdosed content or contaminations [6]. Additionally, consumers of life-style drugs often bypass the healthcare system, so that underlying diseases, such as coronary artery disease, obesity, or anorexia, cannot be detected and pharmacodynamic or pharmacokinetic interactions with other drugs or substances cannot be identified and prevented [5].

Security and encryption experts are continuously working to devise new methods to protect originator drugs from being counterfeited. Thus, secret colour compositions and packaging materials as well as holograms interpretable only with laser readers have been developed to prevent counterfeits from entering the legal supply chain [11]. Yet, counterfeit drugs in developed countries are mainly detected on the illegal pharmaceutical market. Consumers buy medications via the internet to save money or time or because they are too embarrassed about their health problems to seek professional help [5]. The WHO estimates that 50% of medicines bought from online pharmacies that do not list their physical address are counterfeits [12].

The pharmacological content of counterfeit medicines has been examined by both authorities and manufacturers of original products [5]. For example, with PDE5 inhibitors being a main target for counterfeiting, they have been extensively studied [13,14]. A serious incident with counterfeit PDE5 inhibitors occurred in Singapore in 2008, when 4 people died due to hypoglycaemia caused by counterfeits contaminated with glyburide [15].

Microbial contamination and infection are known to be serious risks associated with illegal drug use, the legal use of pharmaceuticals distributed under poor hygienic conditions, and counterfeit medicines for parenteral administration [16-18]. For example, according to a recently published report from Shanghai, China, intravitreal injection of counterfeit bevacizumab contaminated with endotoxin caused acute intraocular inflammation in a series of 80 patients, 21 of whom had to undergo vitrectomy as a result [19]. Whereas parenteral pharmaceuticals must be sterile, non-sterile products may be administered to regions of the human body that are rich in microbial flora and have physical or immunological barriers to infection [20]. However, the US and European pharmacopoeiae state that even in non-sterile preparations, the presence of certain micro-organisms “may have the potential to reduce or even inactivate the therapeutic activity of the product and has a potential to adversely affect the health of the patient. Manufacturers therefore have to ensure a low bioburden of finished dosage forms by implementing current guidelines on Good Manufacturing Practice during the manufacture, storage and distribution of pharmaceutical preparations” [21,22]. Microbiological contamination levels above pharmacopoeial limits may lead to alterations and spoilage of active ingredients and cause adverse effects by infections or toxins.

We here present the results from marketing surveillance studies performed by the Canadian and Austrian official control laboratories between 2008 and 2011 on microbiological contaminations in illegal medicines. Because microbial contamination is a well-known and already widely documented threat for sterile parenteral medicines and because counterfeit and unapproved drugs are frequently sold as solid dosage forms, the main focus of our studies was on solid life-style drugs.

## Methods

All experiments were performed by Health Canada and by the AGES Austrian Medicines and Medical Devices Agency. Both organizations are responsible for market surveillance in their respective countries.

### Canadian study design

Health Canada defines a counterfeit health product as one that is represented as, and likely to be mistaken for, an authentic product [23]. Analyses focused on randomly selected counterfeit and unapproved drugs for the treatment of erectile dysfunction from the illegal market. Twenty-one counterfeit and 31 unapproved PDE5 inhibitors were analysed for microbial contamination. As controls, samples of all available PDE5 inhibitors were obtained from the legal market (Viagra® 25, 50, 100 mg; Cialis® 2.5, 5, 10, 20 mg; Levitra® 5, 10, 20 mg) and analysed. All counterfeit and unapproved samples had been seized by the Royal Canadian Mounted Police or Border Integrity Officers between 2008 and 2010.

The drugs were tested for compliance with the US Pharmacopoeia. A total of five microbiological analyses were performed on the samples: total aerobic microbial count (TAMC), total yeast and mould count (TYMC), pathogens (*Escherichia coli*, *Pseudomonas aeroginosa*, *Salmonella*, and *Staphylococcus aureus*), and enumeration of enterobacteriae and anaerobic bacteria. All analyses, including handling procedures, dilutions, and culture media, were conducted in accordance with the US Pharmacopoeia (USP), Chapters 61 and 62 [24,25], which are harmonized with the European Pharmacopoeia (EP). The assay for sildenafil citrate content was performed according to the corresponding USP monograph.

### Austrian study design

According to the European Medicines Agency, counterfeit medicines are medicines that fail to comply with intellectual-property rights or infringe trademark law [26].

Seven counterfeit PDE5 inhibitors and 26 unapproved medicines (25 solid dosage forms and 1 herbal tea) from the illegal market were randomly selected and analysed for microbial contamination. Unapproved medicines consisted of suspected performance-enhancing drugs or slimming agents (Table 1). As a reference, PDE5 inhibitor products (Viagra® 50 mg; Cialis® 10 mg; Levitra® 10 mg) were obtained from the legal market and examined for microbial contaminations. The drugs had been seized by the Austrian police and the Austrian customs agency between 2008 and 2011. All samples were tested for EP compliance. As in the Canadian study, analyses for TAMC, TYMC, pathogens (*Escherichia coli*, *Pseudomonas aeroginosa*, *Salmonella* and *Staphylococcus aureus*), enterobacteriae, and anaerobic bacteria were performed as applicable. All analyses, including handling procedures, dilutions and culture media, were conducted in accordance with the EP, Chapters 2.6.12, 2.6.13, and 2.6.31 [27-29], which are harmonized with the USP. The assay for sildenafil citrate content was performed according to the corresponding EP monograph.

**Table 1:** Microbiological contamination in counterfeit and unapproved drugs in the Austrian study

**Product**	**Microbial load**		
	**TAMC (CFU/g)**	**TYMC (CFU/g)**	**Pathogens**
**Approved PDE5 inhibitors**			
Sildenafil 50 mg	< 5	< 5	nd
Tadalafil 10 mg	< 5	< 5	nd
Vardenafil 10 mg	< 5	< 5	nd
**Counterfeit PDE5 inhibitors**			
Sildenafil 100 mg #1	< 5	< 5	nd
Sildenafil 100 mg #2	< 5	10	negative
Sildenafil 100 mg #3	< 5	< 5	nd
Sildenafil 100 mg #4	< 5	< 5	nd
Sildenafil 100 mg #5	< 5	< 5	nd
Sildenafil 100 mg #6	170	< 5	negative
Tadalafil 80 mg #7	< 5	< 5	nd
**Other unapproved products**			
Zinc gluconate #8	< 5	< 5	nd
Nicotic acid #9	< 5	< 5	nd
Methandienone #10	80	< 5	negative
Methandienone #11	11 000	< 5	negative
Mephedrone HCl #12	n/a	n/a	n/a
Butylone HCl #13	n/a	n/a	n/a
Methandienone #14	80	60	negative
Stanozolol #15	100	< 5	negative
Stanozolol #16	110	< 5	negative
Clenbuterole 0.02 mg #17	< 5	< 5	nd
Sibutramine, phenolphtalein #18	20	40	negative
Sildenafil 100 mg #19	< 5	< 5	nd
4-Methylethcathinone #20	< 5	< 5	nd
4-Methylcathinone/Coffein #21	< 5	< 5	n/a
4-Methylcathinone/Coffein #22	< 5	< 5	n/a
4-Methylcathinone/Coffein #23	< 5	< 5	nd
3-Fluoromethcathinone/Lidocaine/Coffein #24	< 5	< 5	nd
Coffein/Acetylsalicylic acid #25	< 5	< 5	nd
Slimming herb #26 (herbal product)	720 000	4 000	>10^4^ bile-tolerant gram-negative bacteria

*TAMC*: Total aerobic microbial count; *TYMC*: Total yeast and mould count; *nd*: Not determined; *n/a*: Not applicable (interfering substance prevented successful completion of the test).

Illegal medicines confiscated in Austria were analysed for microbiological contaminations by microbial enumeration tests and tests for specific pathogens.

### Acceptance criteria in the USP and EP

According to the USP and EP, the acceptance criteria for non-aqueous preparations for oral use are 10^3^ colony-forming units (CFU)/g in the TAMC test and 10^2^ CFU/g in the TYMC test. The acceptance criterion for herbal products with cold extraction is 10^5^ CFU/g in the TAMC test, 10^4^ in the TYMC test, and 10^4^ for bile-tolerant gram-negative bacteria.

According to the USP and EP, the acceptance criteria of 10^3^ CFU/g were interpreted as a maximum acceptable count of 2000 CFU/g. The acceptance criterion of 10^5^ CFU/g for herbal products with cold extraction was interpreted as a maximum acceptable count of 500 000 CFU/g.

### Statistical analysis

Due to the skewed distribution of microbiological burden, the non-parametric Wilcoxon rank-sum test, applying the normal approximation, was used to test for differences between medicines in the degree of microbiological contamination. In addition, Fisher’s exact test was used to compare contamination after dichotomization, both with respect to no/any microbiological burden as well as with respect to the acceptance limit of < = 2000 CFU/g versus >2000 CFU/g as defined according to the USP and EP. All statistical tests are presented with two-sided significance levels. The Wilcoxon rank-sum test comparing microbiological contamination of legal versus illegal (counterfeit and unapproved) medicines was considered the primary analysis.

## Results

### Only counterfeit and unapproved PDE inhibitors showed increased contamination

**Figure 1:**
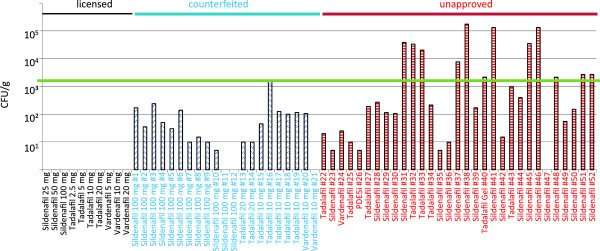
**Microbiological load in PDE5 inhibitors in Canada.** Products from the legal market (black), counterfeit (blue), and unapproved (red) drugs were tested for microbiological contamination. The pharmacopoeial limits of 10^3^ CFU (2000 counts)/g are indicated by the green line.

Not a single CFU was detected in the approved PDE5 inhibitor products obtained through the legal pharmaceutical supply chain—neither in the Canadian nor in the Austrian study. Thus, although the USP and EP allow an upper limit of 10^3^ CFU/g, no cultivable microbial contaminations were detected for these pharmaceuticals produced under controlled GMP conditions (Figure 1).

In the Canadian study, 12 of the 31 unapproved PDE5 inhibitor samples (39%) were contaminated with more than 10^3^ CFU/g (Figure 1). Taking counterfeit and unapproved drugs together, 12 of the 52 samples (23%) were contaminated with more than 10^3^ CFU/g, 36 samples (69%) showed increased levels of microbial contamination that were within the acceptable limits, and only 4 of the 52 illegal products (8%) showed excellent results with no cultivable contamination.

In the Austrian study, none of the 7 counterfeit PDE5 inhibitor samples tested showed a microbial contamination above the EP limit. Contamination with colony-forming microorganisms within EP limits was found in 2 of the 7 samples (29%, Table 1).

### Statistical analysis of increased microbiological burden in counterfeit and unapproved PDE5 inhibitors in Canada

Wilcoxon rank-sum and Fisher’s exact tests were performed to test for statistical significance of observed differences between legal and illegal (counterfeit and unapproved) PDE5 inhibitors in Canada. In the Wilcoxon rank-sum test, the degree of microbiological contamination (CFU/g) in illegal medicines was significantly higher than in the legal products (p < 0.0001, two-sided). The Fisher’s exact test demonstrated that the number of contaminated illegal samples (>0 CFU/g; as opposed to no contamination at all) was statistically significantly higher than in legal samples (p < 0.0001, two-sided). Overall, therefore, both the number of cultivable contaminations (Wilcoxon rank-sum test) and the number of contaminated samples (>0 CFU/g; Fisher’s exact test) were significantly higher among illegal PDE5 inhibitors.

Comparison for non-compliance with the pharmacopoeia limits (>2000 CFU/g) did not show a statistically significant difference between illegal and legal medicines, but a clear trend was observed (p = 0.1864, two-sided Fisher’s exact test). Unapproved medicines showed a clear statistically significant increase of non-compliance when compared to the counterfeit products (p < 0.0001, two-sided Fisher’s exact test).

Due to the limited number of PDE5 inhibitors from Austria, statistical analysis was only performed for the Canadian market.

### *Bacillus* contaminations were frequent in counterfeit and unapproved PDE5 inhibitors

The identified species are summarized in Table 2. None of the pathogens specifically defined in the pharmacopeiae were detected. Amongst others, mainly contaminations with *Bacillus* ssp. were observed. Identified *Bacillus* species included *B. firmus, B. lentus, B. megaterium, B. pumilus, B. polymyxa, B. subtillis/amyloliquefaciens/atrophaeus, B. licheniformis, B. cereus/thuringensis/mycoides, B. pumilus, B. coagulans, B. fusiformis, B. circulans,* and *B. glucanolyticus*.

**Table 2:** Identified bacterial species in counterfeit and illegal PDE inhibitors in the Canadian study

**Product**	**Identified bacteria**
**Approved PDE5 inhibitors**	
Sildenafil 25 mg	n/a
Sildenafil 50 mg	n/a
Sildenafil 100 mg	n/a
Tadalafil 2.5 mg	n/a
Tadalafil 5 mg	n/a
Tadalafil 10 mg	n/a
Tadalafil 20 mg	n/a
Vardenafil 5 mg	n/a
Vardenafil 10 mg	n/a
Vardenafil 20 mg	n/a
**Counterfeit PDE5 inhibitors**	
Sildenafil 100 mg #1	*Bacillus spp.*
Sildenafil 100 mg #2	*Serratia spp., Paenibacillus polymyxa, Paenibacillus amylolyticus, Bacillus ssp., Paenibacillus gluconolyticus, Corynebacterium spp., Brevibacillus borstelensis*
Sildenafil 100 mg #3	*Brevibacillus choshinensys, Bacillus spp.*
Sildenafil 100 mg #4	*Bacillus ssp.*
Sildenafil 100 mg #5	*Bacillus spp.*
Sildenafil 100 mg #6	*Bacillus ssp., Brevibacillus choshinensys, Virgibacillus pantothenticus, Alicyclobacillus acidoterrestris, Sphingomonas paucimobilis, Paenibacillus polymyxa, Propionibacterium acnes, Brevibacillus borstelensis, Bacillus spp.*
Sildenafil 100 mg #7	*Micrococcus luteus*
Sildenafil 100 mg #8	*Bacillus ssp., Propionibacterium acnes*
Sildenafil 100 mg #9	*Paenibacillus amylolyticus*
Sildenafil 100 mg #10	*Brevibacillus choshinensys*
Sildenafil 100 mg #11	n/a
Sildenafil 100 mg #12	n/a
Tadalafil 20 mg #13	*Bacillus ssp.*
Tadalafil 20 mg #14	*Kocuria rosea, Bacillus pumilus*
Tadalafil 10 mg #15	*Bacillus spp*
Tadalafil 10 mg #16	*Bacillus spp*
Tadalafil 10 mg #17	*Granulicatella adiacens, Streptococcus salivarus, Bacillus spp*
Tadalafil 10 mg #18	*Bacillus spp*
Tadalafil 10 mg #19	*Bacillus spp*
Vardenafil 20 mg #20	*Bacillus spp*
Vardenafil 20 mg #21	n/a
**Unapproved PDE5 inhibitors**	
Tadalafil #22	*Paenibacillus lautus, Paenibacillus durus, Paenibacillus glucanolyticus, Bacillus spp.*
Sildenafil #23	*Bacillus ssp., Paenibacillus spp.*
Vardenafil #24	*Bacillus ssp.*
Tadalafil #25	*Staphylococcus epidermidis, Bacillus spp.*
PDE5 inhibitor #26	*Bacillus spp.,*
Tadalafil #27	*Bacillus spp.*
Sildenafil #28	*Bacillus spp.*
Sildenafil #29	*Bacillus spp.*
Sildenafil #30	*Bacillus spp., Sphingomonas paucimobilis, aerobic Actinomycetes*
Sildenafil #31	*Bacillus spp.*
Tadalafil #32	*Bacillus spp.*
Tadalafil #33	*Bacillus spp.*
Tadalafil #34	*Granulicatella adiacens, Streptococcus salivarus, Bacillus spp.*
Sildenafil #35	*Bacillus spp.*
Sildenafil #36	*Bacillus spp., Micrococcus spp.*
Sildenafil #37	*Bacillus spp.*
Sildenafil #38	*Bacillus spp.*
Sildenafil #39	*Bacillus spp.*
Tadafenil #40	*Bacillus spp.*
Sildenafil #41	*Bacillus spp.*
Sildenafil #42	*Bacillus spp.*
Tadafenil #43	*Bacillus spp., Staphylococcus hominis*
Sildenafil #44	*Bacillus spp.*
Sildenafil #45	*Bacillus spp.*
Sildenafil #46	*Bacillus spp.*
Sildenafil #47	*Bacillus spp.*
Sildenafil #48	*Bacillus spp.*
Sildenafil #49	*Bacillus spp.*
Sildenafil #50	*Bacillus spp.*
Sildenafil #51	*Bacillus spp.*
Sildenafil #52	*Bacillus spp.*

*n/a*: Not applicable.Bacterial species identified in the contaminated samples of Figure 1.

### Inconsistent doses of active pharmaceutical ingredients in counterfeit PDE5 inhibitors

Although this was not the main focus of our study, we also examined the content of active ingredients in the counterfeit samples (Additional file 1). Of all 26 counterfeits tested, 24 (92%) did not contain the labelled amount of PDE5 inhibitor (acceptance criterion ± 10% of the labelled amount). In 21 samples (81%), a reduced content of the active ingredient was detected, whereas 3 samples (12%) were about 2-fold over-dosed. Interestingly, 14 of the 26 samples (54%) also contained trace amounts of a second PDE5 inhibitor.

### Microbiological contamination in other product classes tested

When examining the 25 non-PDE5-inhibitor unapproved medicines with solid dosage forms from the Austrian market, one product containing methandienone was contaminated with 1.1 × 10^4^ CFU/g in the TAMC test, thus exceeding the EP limit of 10^3^ CFU/g. Of the 25 unapproved medicines, one (4%) did not comply with EP standards and an additional 7 (28%) showed a microbial load higher than that seen in the legal products manufactured under defined GMP conditions.

### Increased microbiological burden in an unapproved herbal product

One unapproved herbal medicinal product was tested in Austria. The product contained brown-coloured dried plant tissue and was contaminated with 720 000 CFU/g in the TAMC test (Table 1), exceeding the acceptance criterion of 10^5^ CFU/g (maximum of 500 000 counts). Besides, the sample also exceeded the EP limit of 10^4^ CFU/g of bile-tolerant gram-negative bacteria. *Klebsiella pneumonia* was identified as the prevalent bile-tolerant species.

## Discussion

The microbiological content in non-sterile products has to be controlled to a level consistent with patient safety [20]. Microbial enumeration tests are required to demonstrate production under acceptable hygienic conditions. Whenever pharmacopoeial limits are exceeded, the microbiological quality of manufacturing was not sufficiently controlled and adverse effects on product and patient safety cannot be excluded. Additionally, according to the US and European pharmacopoeiae, the significance of recovered microorganisms must be evaluated and the absence of specific pathogens demonstrated depending on the route of administration [21,22]. Microbiological contaminations may be introduced by the raw material, through the manufacturing process, or during packaging and transport, and risk-based control points should be incorporated into the manufacturing process [20].

Our data, derived from independent studies performed in two different pharmaceutical markets, confirm that counterfeit and unapproved medicines are not manufactured under the same hygienic condition as genuine products. The Canadian and Austrian studies presented both showed that none of the PDE5 inhibitors from the legitimate supply chain produced under GMP conditions contained any microbial burden detectable by routine pharmacopoeial testing, whereas 92% of Canadian and 29% of Austrian PDE5 inhibitor samples seized from the illegal market contained cultivable contaminations. Also, 23% of counterfeit and unapproved PDE5 inhibitors seized in Canada and 6% of all tested samples from the Austria illegal market failed to comply with the pharmacopoeial limits in microbial enumeration tests.

In the Canadian study, the microbiological contaminations in unapproved products were higher than in counterfeit products. It may be hypothesized that individuals producing counterfeit products may be more intent on using reasonably safe production conditions than those trading in unapproved products. However, the difference between counterfeit and unapproved products seen in our study may not be generalizable. Thus, we found that both product groups contained higher amounts of microbiological contaminations than products from the legal market, indicating that both groups may be expected to be prepared under less hygienic production conditions than genuine medicinal products. In the Austrian study, a higher rate of contaminations above EP limits was also observed for unapproved than in counterfeit products, but due to the different nature of the products tested in Austria, direct statistical comparison was not performed.

Most identified contaminating bacteria in PDE5 inhibitors belong to the *Bacillus* genus. *Bacillus ssp*. can form resistant endospores and are part of the normal environmental flora [30]. This may be indicative of contamination by human manipulators. Although no major pathogens were isolated, some of the identified *Bacillus species* are capable of causing human infection [30]. Besides, *Serratia* species, *Propionibacterium acnes, Granulicatella adiacens, Streptococcus salivarus, Staphylococcus epidermidis, Staphylococcus hominis, Enterococcus gallinarum,* and *Sphingomonas paucimobilis* were identified and have the potential to cause clinical symptoms of infection [31,32]. In view of the oral route of administration of PDE5 inhibitors, the bacterial species detected may not pose an increased risk to consumers, provided that the total counts do not exceed pharmacopoeial limits.

*Klebsiella pneumoniae* was identified in an unapproved herbal medicinal tea, with the included package leaflet recommending extraction with cold water. This method of extraction may not be expected to reduce viable contaminations. *K. pneumoniae* is a gram-negative bacterium that can cause bacterial pneumonia and hospital-acquired urinary tract and wound infections [33]*. K. pneumoniae* mainly attacks immunocompromised patients and individuals with underlying diseases, such as diabetes mellitus [33]. The examined infusion failed to comply with the EP limits for bile-tolerant gram-negative bacteria and for total bacterial counts (TAMC). Although the concentrations of *K. pneumoniae* detected in this product are unlikely to lead to clinical infection in healthy humans [34], a potential threat cannot be excluded, especially when storage conditions are not monitored and further bacterial growth might occur.

Two potential threats arise from microbiological contaminations in pharmaceutical products. First, certain microorganisms may alter the quality of the active ingredients and even lead to spoilage of the product. Second, microbiological contaminations may directly cause adverse effects by producing toxins or causing infections. Product alterations are less likely in solid PDE5 inhibitors but may occur in herbal products. In general, the threats from microbiological contaminations are higher for herbal products and products with a moisture content that supports bacterial survival and growth than for solid dosage forms. Accordingly, a recent study found high levels of bacterial contaminations in counterfeit toothpaste [35], and an outbreak of *Salmonella montevideo* has been associated with a dietary herbal food supplement [36]. Herbal products show higher levels and limits of contamination because of the raw materials they contain and the mild production methods used. In contrast, PDE5 inhibitors are less likely to be contaminated with bacteria due to the synthetisation process of the active substances, the chemicals used, and the low moisture content. Even in synthesised medicines, however, contaminations by excipients or cutting agents and human manipulation during manufacture and transport pose a risk when hygienic production conditions are not guaranteed. In recent years, several cases of injectional anthrax most likely caused by contaminated heroin have been described in Europe [37]. Suggested routes of contaminations included animal-derived sources, such as bone meal or animal hides [38]. Although this is clearly a worst-case scenario associated with injectional administration of an illicit drug, it illustrates that the lack of controls during manufacturing and/or transport of pharmaceutical active substances can lead to contamination with severe pathogens. Interestingly, despite good evidence for the cause of infection, neither *B. anthracis* nor its genome was detected in any of the heroin samples tested [37]. The production of illegal drugs and illegal pharmaceuticals might differ, but for both, in the absence of strictly quality-controlled hygienic production conditions, even testing for specific pathogens may not be sufficient to detect serious contaminations.

## Conclusions

Based on our studies, it may be assumed that various groups of illegal medications contain increased levels of microbial contaminations. Our results demonstrate that illegal pharmaceuticals are produced under less hygienic conditions than legitimate products manufactured under controlled and defined GMP conditions.

To gain broader insights into the microbiological burden in counterfeit drugs, we recommend the risk-based inclusion of microbiological quality studies in the surveillance of the illegal pharmaceutical market.

## Competing interests

The authors declare that they have no competing interests.

## Authors’ contributions

All authors read and approved the final manuscript. DP collected and analysed the data and drafted the article. YLT and CT analysed the results, drafted the study report by Health Canada, and critically revised the article. JB and JG planned and designed the Canadian study part, analysed the Canadian results, and critically revised the article. AH, HS, GB, and AM planned and designed the Austrian part of the study and analysed the data. BG performed the experiments and analysed the results. CG performed the statistical analyses of the results and critically revised the article.

## Pre-publication history

The pre-publication history for this paper can be accessed here:

http://www.biomedcentral.com/2050-6511/15/34/prepub

## Supplementary Material

Additional file 1 **PDE5 inhibitor content in counterfeit drugs from Canada and Austria.** Inconsistent doses of active pharmaceutical ingredients were detected in counterfeit PD5 inhibitors. Click here for file

## References

[B1] World Health Organization Growing threat from counterfeit medicines Bull World Health Organ 2010 88 4 247 248 20431784 10.2471/BLT.10.020410 PMC2855605

[B2] Mackey TK Liang BA Improving global health governance to combat counterfeit medicines: a proposal for a UNODC-WHO-Interpol trilateral mechanism BMC Med 2013 11 233 10.1186/1741-7015-11-233 24228892 PMC4225602

[B3] Gostin LO Buckley GJ Kelley PW Stemming the global trade in falsified and substandard medicines JAMA 2013 309 16 1693 1694 10.1001/jama.2013.3048 23579391

[B4] United Nations Office on Drugs and Crime Transnational trafficking and the rule of law in West Africa: a threat assessment 2009 https://www.unodc.org/documents/data-and-analysis/Studies/West_Africa_Report_2009.pdf (accessed on 2013-02-12)

[B5] Jackson G Patel S Khan S Assessing the problem of counterfeit medications in the United Kingdom Int J Clin Pract 2012 66 3 241 250 10.1111/j.1742-1241.2011.02826.x 22070229

[B6] Jackson G Faking it: the dangers of counterfeit medicine on the internet Int J Clin Pract 2009 63 2 181 10.1111/j.1742-1241.2008.01989.x 19196352

[B7] World Health Organization General information on counterfeit medicines http://www.who.int/medicines/services/counterfeit/overview/en/ (accessed 2013-02-04)

[B8] Jung C Evaluating FDA Communications to Reduce Counterfeit and Unapproved Drugs in the Clinical Setting Risk Communication Advisory Committee Meeting 2013 http://www.fda.gov/downloads/AdvisoryCommittees/CommitteesMeetingMaterials/RiskCommunicationAdvisoryCommittee/UCM370232.pdf (accessed 2013-11-29)

[B9] U.S. Food and Drug Administration Letters to doctors about risks of purchasing unapproved versions of botox and other medications from foreign or unlicensed suppliers http://www.fda.gov/Drugs/DrugSafety/DrugIntegrityandSupplyChainSecurity/ucm330610.htm, (accessed 02-12-2013)

[B10] U.S. Food and Drug Administration Fraudulent versions of botox found in the United States http://www.fda.gov/drugs/drugsafety/ucm349503.htm , accessed 02-12-2013

[B11] Schubert C New countermeasures considered as drug counterfeiting grows Nat Med 2008 14 7 700 10.1038/nm0708-700b 18607355

[B12] Mackey TK Liang BA The global counterfeit drug trade: patient safety and public health risks J Pharm Sci 2011 100 11 4571 4579 10.1002/jps.22679 21698604

[B13] Jackson G Arver S Banks I Stecher VJ Counterfeit phosphodiesterase type 5 inhibitors pose significant safety risks Int J Clin Pract 2010 64 4 497 504 10.1111/j.1742-1241.2009.02328.x 20088883 PMC3069491

[B14] Low MY Zeng Y Li L Ge XW Lee R Bloodworth BC Koh HL Safety and quality assessment of 175 illegal sexual enhancement products seized in red-light districts in Singapore Drug Saf 2009 32 12 1141 1146 10.2165/11316690-000000000-00000 19916581

[B15] Kao SL Chan CL Tan B Lim CC Dalan R Gardner D Pratt E Lee M Lee KO An unusual outbreak of hypoglycemia N Engl J Med 2009 360 7 734 736 10.1056/NEJMc0807678 19213693

[B16] Liang BA Fade to black: importation and counterfeit drugs Am J Law Med 2006 32 2–3 279 323 16927613 10.1177/009885880603200207

[B17] Kaushik KS Kapila K Praharaj AK Shooting up: the interface of microbial infections and drug abuse J Med Microbiol 2011 60 Pt 4 408 422 21389334 10.1099/jmm.0.027540-0

[B18] Mugoyela V Mwambete KD Microbial contamination of nonsterile pharmaceuticals in public hospital settings Ther Clin Risk Manag 2010 6 443 448 20957135 10.2147/TCRM.S12253 PMC2952482

[B19] Wang F Yu S Liu K Chen FE Song Z Zhang X Xu X Sun X Acute intraocular inflammation caused by endotoxin after intravitreal injection of counterfeit bevacizumab in Shanghai China Ophthalmology 2013 120 2 355 361 10.1016/j.ophtha.2012.07.083 23084126

[B20] U.S. Pharmacopoeial Convention Bioburden Control of Non-sterile Drug Substances and Products - Draft United States Pharmacopoeia 2013 Rockville: USP 39 (4) In-Process Revision, U.S. Pharmacopoeial Convention

[B21] U.S. Pharmacopoeial Convention Microbiological Examination of non-sterile Products: Acceptance Criteria for pharmaceutical Preparations and Substances for pharmaceutical use United States Pharmacopoeia 2012 Rockville: General Chapter 1111((USP 35): 1733), U.S. Pharmacopoeial Convention

[B22] European Directorate for the Quality of Medicines & HealthCare Microbiological Quality of non-sterile Pharmaceutical Preparations and Substances for Pharmaceutical use European Pharmacopoeia 70 2011 Strasbourg: Chapter 5.1.4, EDQM

[B23] Health Canada Policy on Counterfeit Health Products POL-0048 2010 Ottawa, Ontario: Policy and Strategic Planning Division, Health Products and Food Branch Inspectorate

[B24] U.S. Pharmacopoeial Convention Microbiological examination of nonsterile products: microbiological enumeration tests United States Pharmacopeia 2012 Rockville: General Chapter 61(USP 35), U.S. Pharmacopoeial Convention 56 60

[B25] U.S. Pharmacopoeial Convention Microbiological examination of nonsterile products: tests for specified microorganisms United States Pharmacopeia 2012 Rockville: General Chapter 62 (USP 35), U.S. Pharmacopoeial Convention 60 61

[B26] European Medicines Agency Falsified medicines EMA Homepage, http://www.emea.europa.eu/ema/index.jsp?curl=pages/special_topics/general/general_content_000186.jsp&mid=WC0b01ac058002d4e8. (accessed 2013-04-26)

[B27] European Directorate for the Quality of Medicines & HealthCare Microbiological examination of non-sterile products: microbial enumeration tests European Pharmacopoeia 70 2011 Strasbourg: Chapter 2.6.12, EDQM 163 167

[B28] European Directorate for the Quality of Medicines & HealthCare Microbiological examination of non-sterile products: test for specified micro-organisms European Pharmacopoeia 70 2011 Strasbourg: Chapter 2.6.13, EDQM 167 171

[B29] European Directorate for the Quality of Medicines & HealthCare Microbiological examination of herbal medicinal products for oral use European Pharmacopoeia 70 2011 Strasbourg: Chapte 2.6.31, EDQM 197 198

[B30] Ozkocaman V Ozcelik T Ali R Ozkalemkas F Ozkan A Ozakin C Akalin H Ursavas A Coskun F Ener B Tunali A Bacillus spp. among hospitalized patients with haematological malignancies: clinical features, epidemics and outcomes J Hosp Infect 2006 64 2 169 176 10.1016/j.jhin.2006.05.014 16891037

[B31] American Society For Microbiology Manual of Clinical Microbiology 2011 10 Washington DC: ASM Press Editor in Chief: James Versalovic

[B32] Mandell GL Bennett JE Dolin R Principles and Practice of Infections and Diseases 2010 7 Churchill Livingstone, Elsevier, Philadelphia: Imprint

[B33] Podschun R Ullmann U Klebsiella spp. as nosocomial pathogens: epidemiology, taxonomy, typing methods, and pathogenicity factors Clin Microbiol Rev 1998 11 4 589 603 9767057 10.1128/cmr.11.4.589 PMC88898

[B34] Public Health Agency of Canada Klebsiella ssp. pathogen safety datasheet - infectious substances http://www.phac-aspc.gc.ca/lab-bio/res/psds-ftss/klebsiella-eng.php (accessed 2014-04-16)

[B35] Brzezinski JL Craft DL Characterization of microorganisms isolated from counterfeit toothpaste J Forensic Sci 2012 57 5 1365 1367 10.1111/j.1556-4029.2012.02130.x 22509777

[B36] Stocker P Rosner B Werber D Kirchner M Reinecke A Wichmann-Schauer H Prager R Rabsch W Frank C Outbreak of Salmonella Montevideo associated with a dietary food supplement flagged in the Rapid Alert System for Food and Feed (RASFF) in Germany, 2010 Euro Surveill 2011 16 50 20040 22221497

[B37] Hanczaruk M Reischl U Holzmann T Frangoulidis D Wagner DM Keim PS Antwerpen MH Meyer H Grass G Injectional anthrax in heroin users, europe, 2000–2012 Emerg Infect Dis 2014 20 2 322 323 10.3201/eid2002.120921 24447525 PMC3901468

[B38] Price EP Seymour ML Sarovich DS Latham J Wolken SR Mason J Vincent G Drees KP Beckstrom-Sternberg SM Phillippy AM Koren S Okinaka RT Chung WK Schupp JM Wagner DM Vipond R Foster JT Bergman NH Burans J Pearson T Brooks T Keim P Molecular epidemiologic investigation of an anthrax outbreak among heroin users, Europe Emerg Infect Dis 2012 18 8 1307 1313 10.3201/eid1808.111343 22840345 PMC3414016

