# Supplementary material for: Microbiological contamination in counterfeit and unapproved drugs
Source: BMC Pharmacol Toxicol. 2014 Jun 26;15:34. doi: 10.1186/2050-6511-15-34 (PMC4088308; doi:10.1186/2050-6511-15-34)
Supplement: Additional file 1 — PDE5 inhibitor content in counterfeit drugs from Canada and Austria. Inconsistent doses of active pharmaceutical ingredients were detected in counterfeit PD5 inhibitors. [file 2050-6511-15-34-S1.docx]

| **Labeled content** | **Detected content** |
| --- | --- |
| **Counterfeit PDE5i Canada** | |
| Sildenafil 100mg #1 | *Sildenafil 29 mg/tablet, Tadalafil 0.4 mg/tablet* |
| Sildenafil 100mg #2 | *Sildenafil 35 mg/tablet, Tadalafil 0.1 mg/tablet* |
| Sildenafil 100mg #3 | *Sildenafil 18 mg/tablet, Tadalafil 0.4 mg/tablet* |
| Sildenafil 100mg #4 | *Sildenafil 68 mg/tablet* |
| Sildenafil 100mg #5 | *Sildenafil 34 mg/tablet, Tadalafil 0.1 mg/tablet* |
| Sildenafil 100mg #6 | *Sildenafil 12 mg/tablet, Tadalafil 1mg/tablet* |
| Sildenafil 100mg #7 | *Sildenafil 58 mg/tablet, Tadalafil 1 mg/tablet* |
| Sildenafil 100mg #8 | *Sildenafil 54 mg/tablet, Tadalafil 0.3 mg/tablet* |
| Sildenafil 100mg #9 | *Sildenafil 67 mg/tablet, Tadalafil 0.05 mg/tablet* |
| Sildenafil 100mg #10 | *Sildenafil 55 mg/tablet, Tadalafil 0.2 mg/tablet* |
| Sildenafil 100mg #11 | *Sildenafil 51 mg/tablet, Tadalafil 0.5 mg/tablet* |
| Sildenafil 100mg #12 | *Sildenafil 46 mg/tablet, Tadalafil 0.6 mg/tablet* |
| Tadalafil 20mg #13 | *Tadalafil 12 mg/tablet, Sildenafil 0.2 mg/tablet* |
| Tadalafil 20mg #14 | *Tadalafil 11 mg/tablet, Sildenafil 0.2 mg/tablet* |
| Tadalafil 10mg #15 | *Tadalafil 10 mg/tablet* |
| Tadalafil 10mg #16 | *Tadalafil 9.5 mg/tablet* |
| Tadalafil 10mg #17 | *Tadalafil 20 mg/tablet* |
| Tadalafil 10mg #18 | *Tadalafil 19 mg/tablet* |
| Tadalafil 10mg #19 | *Tadalafil 20 mg/tablet* |
| Vardenafil 20mg #20 | *Microbial testing only* |
| Vardenafil 20mg #21 | *Microbial testing only* |
| **Counterfeit PDE5i Austria** | |
| Sildenafil 100 mg #1 | *Sildenafil 64 mg/tablet* |
| Sildenafil 100 mg #2 | *Sildenafil 66 mg/tablet* |
| Sildenafil 100 mg #3 | *Sildenafil 64 mg/tablet* |
| Sildenafil 100 mg #4 | *Sildenafil 84 mg/tablet* |
| Sildenafil 100 mg #5 | *Sildenafil 85 mg/tablet* |
| Sildenafil 100 mg #6 | *Sildenafil 89 mg/tablet* |
| Tadalafil 80 mg #7 | *Sildenafil 0.59 mg/tablet*,*Tadalafil 20.74 mg/tablet* |
